# Computational and Experimental Characterization of Aligned Collagen across Varied Crosslinking Degrees

**DOI:** 10.3390/mi15070851

**Published:** 2024-06-29

**Authors:** Shengmao Lin, Nashaita Y. Patrawalla, Yingnan Zhai, Pengfei Dong, Vipuil Kishore, Linxia Gu

**Affiliations:** 1 School of Civil Engineering and Architecture, Xiamen University of Technology, Xiamen 361024, China; 2 Department of Biomedical Engineering and Science, Florida Institute of Technology, Melbourne, FL 32901, USApdong@fit.edu (P.D.); vkishore@fit.edu (V.K.); 3 Department of Chemistry and Chemical Engineering, Florida Institute of Technology, Melbourne, FL 32901, USA

**Keywords:** collagen, tissue engineering, electrochemical alignment, genipin crosslinking, finite element modeling, FEM, RVE, Voronoi network

## Abstract

Collagen-based scaffolds have been widely used in tissue engineering. The alignment of collagen fibers and the degree of crosslinking in engineering tissue scaffolds significantly affect cell activity and scaffold stability. Changes in microarchitecture and crosslinking degree also impact the mechanical properties of collagen scaffolds. A clear understanding of the effects of collagen alignment and crosslinking degrees can help properly control these critical parameters for fabricating collagen scaffolds with desired mechanical properties. In this study, combined uniaxial mechanical testing and finite element method (FEM) were used to quantify the effects of fiber alignment and crosslinking degree on the mechanical properties of collagen threads. We have fabricated electrochemically aligned collagen (ELAC) and compared it with randomly distributed collagen at varying crosslinking degrees, which depend on genipin concentrations of 0.1% or 2% for crosslinking durations of 1, 4, and 24 h. Our results indicate that aligned collagen fibers and higher crosslinking degree contribute to a larger Young’s modulus. Specifically, aligned fiber structure, compared to random collagen, significantly increases Young’s modulus by 112.7% at a 25% crosslinking degree (0.1% (4 h), i.e., 0.1% genipin concentration with a crosslinking duration of 4 h). Moreover, the ELAC Young’s modulus increased by 90.3% as the crosslinking degree doubled by changing the genipin concentration from 0.1% to 2% with the same 4 h crosslinking duration. Furthermore, verified computational models can predict mechanical properties based on specific crosslinking degrees and fiber alignments, which facilitate the controlled fabrication of collagen threads. This combined experimental and computational approach provides a systematic understanding of the interplay among fiber alignment, crosslinking parameters, and mechanical performance of collagen scaffolds. This work will enable the precise fabrication of collagen threads for desired tissue engineering performance, potentially advancing tissue engineering applications.

## 1. Introduction

Collagen is the primary structural protein in most extracellular matrix of the human body, and it contributes to its wide applicability for various tissue engineering and regenerative medicine applications. Collagen, as a biomaterial, is known for its excellent cytocompatibility, hydrophilicity, low antigenicity, and biodegradability. However, it often lacks the mechanical properties necessary for biomedical applications [1,2]. One approach for enhancing the mechanical properties of collagen scaffolds is by introducing an anisotropic orientation of collagen fibers that mimic the native tissue microarchitecture, thereby providing superior mechanical properties together with critical biophysical factors to guide tissue-specific cell response [3,4,5]. Of the different biofabrication methods that exist to align collagen fibers, the electrochemical alignment of collagen (ELAC) technique is one promising approach often used to deliver densely packed and highly aligned collagen scaffolds [6,7]. In this process, dialyzed collagen solution is extruded between two electrodes and exposed to a low-level electric field, which triggers the formation of a pH gradient between the wire electrodes. As the pH gradient develops, the collagen molecules acquire a charge and repel away from the electrodes, aligning along the isoelectric point where the net charge is zero [6]. Consequently, this process results in the formation of a collagen thread composed of highly aligned collagen fibers. Prior work has confirmed that the integrity of collagen is retained after electrochemical alignment, and the collagen is not denatured by the process [6,8]. The aligned collagen fiber structures affect cell activity and the physical properties of scaffolds, such as swelling capacity and stability, in tissue engineering [7]. Cells were oriented along the longitudinal direction that parallelled the direction of collagen fiber alignment [7,9]. Better cell activity, including cell proliferation, adhesion, and differentiation, was afforded by the aligned collagen fiber structure [7,10,11,12] because cells are stimulated by the mechanical traction of oriented fibers through contact induction [11,13]. ELAC threads can be braided in a facile fashion to yield 3D-aligned collagen scaffolds with porous architecture for use in tendon tissue engineering applications [14]. Further, the process allows for the addition of secondary components (e.g., decorin, Bioglass, elastin) to generate composite materials that better mimic the compositional properties of native tissue [6,8,15,16]. Also, the scaffold with aligned collagen fiber showed a higher swelling ratio and higher residual mass after 24 h, which indicates the advantages in stability and potential drug delivery for tissue engineering [7,17].

In addition to collagen alignment, various crosslinking techniques have been employed to enhance the mechanical properties and stability of collagen scaffolds. Physical crosslinking methods such as UV irradiation, gamma irradiation, and dehydrothermal treatment offer simplicity and versatility in tissue engineering applications, albeit with limited control over crosslinking degree and non-uniform mechanical properties [18,19]. Chemical interaction-based methods utilize compounds like aldehydes, carbodiimides, and isocyanates to yield robust collagen scaffolds, but some of them are cytotoxic [19,20]. Genipin, derived from a naturally occurring source of the Gardenia fruit, is an alternative chemical crosslinker that forms stable crosslinks between collagen molecules to enhance the mechanical properties of collagen scaffolds while exhibiting low cytotoxicity and maintaining biocompatibility [21,22]. Modulating the genipin crosslinking parameters, such as genipin concentration and crosslinking duration, has been shown to deliver ELAC threads with tunable mechanical properties [16,23]. Genipin crosslinked ELAC thread with higher crosslinking degree showed a higher fold increase in cell number, which indicated the scaffold represents good cytocompatibility for tissue engineering. Furthermore, the scaffold has delivered mechanical properties comparable to that of native tendon tissue [23]. The optimal crosslinked ELAC threads using genipin can be a prospective engineered tissue for tendon healing and replacement, which can be combined with 3D extrusion bioprinting methods [24] or 4D printing, introducing the time dimension for microstructural changes [25].

Mechanical properties are increasingly being regarded as important design factors for engineering tissue. The Young’s modulus of engineering tissue has been reported to influence cell activities. Engler et al. demonstrated that mesenchymal stem cells (MSCs) selectively differentiate into neurons, myoblasts, or osteoblasts on the substrate with various elasticities [26]. Wang et al., have shown that the stiffness of engineering tissue substrate and matrix regulates cell physiological processes, including cell adhesion and proliferation [27]. Meanwhile, the topography of ELAC threads was observed to stimulate tenogenic differentiation of human MSCs [12]. However, the effect of ELAC deformation under tensile load on MSCs differentiation has not been well discussed. And the deformation of ELAC fibers shall be studied on the micro or nanoscale.

Diverse mechanical testing approaches have been used to evaluate the mechanical properties of the aligned collagen scaffolds, including the uniaxial tensile test [28], the biaxial tensile test [29], and the nanoindentation test [30]. Previous studies illustrated that the mechanical properties of ELAC threads are highly related to several factors, including collagen fiber orientation, isoelectric focusing voltage and duration, and collagen concentrations [31]. Furthermore, the crosslinking degree is another important factor that affects the mechanical properties of ELAC threads [23]. However, the application of uniaxial tensile tests on bulk ELAC scaffold cannot measure the mechanical effect due to the micro or nanoscale deformations that take place between chemically crosslinked collagen fibers. The computational simulation method can be a solution to evaluate the mechanical behavior of crosslinked parts under the effect of varied collagen alignment and crosslinking degree.

Recently, computational simulation with finite element method (FEM) has been employed to predict the behavior of collagen scaffolds. Xu et al. applied constitutive modeling to capture the stress–strain relationship of collagen gels with collagen fiber dispersion and orientation parameters based on strain energy function [29]. The crosslinking degree variations were represented by stress-like parameters [29]. In a two-dimensional model, the crosslinked fibers were constrained as welded joints [32] or pin joints with free rotation [33]. The three-dimensional models for randomly aligned fibers were built to investigate the interaction between the mechanical properties of the fiber network and the cultured cells’ dynamic behaviors [34,35,36]. The crosslinked fibers in three-dimensional networks were considered regular fibers [34] or torsional springs, and the rotational property was based on experimental results [35]. The effect of crosslinking parts on the scaffold’s mechanical properties has not been well explained in previous models. With the Voronoi network, the fibrous structure is directly constructed by beam elements [37,38]. The crosslinked parts of fibers are randomly joined without artifact modifications [39], which are flexible in bending, stretching, twisting, and shear modes of deformation [40]. The Voronoi model provides a closer approximation to the collagen fibers crosslinked microstructures.

The goal of this study is to combine mechanical tests and FEM to determine and predict the mechanical properties of collagen threads across various crosslinking degrees and fiber alignments. Experimental data from uniaxial tension tests and Scanning Electron Microscopy (SEM) were used to characterize the collagen microstructure and mechanical properties. The effect on collagen threads’ mechanical properties was determined based on the variation of collagen fiber alignment, genipin concentration, and crosslinking duration. Computational simulations were performed by developing the representative volume element (RVE) models to discuss the effect of fiber alignment and crosslinking degree on collagen threads’ mechanical properties. The computer models, once validated by the experimental data, were used to predict the properties at particular crosslinking degrees and fiber alignments. The prediction model developed in this work provides a systematic understanding of the intricate interplay between crosslinking parameters and mechanical performance in ELAC scaffolds.

## 2. Materials and Methods

### 2.1. Materials

Acid-solubilized type I collagen (PureCol, 3.1 mg/mL in 10 mM HCl) was purchased from Advanced Biomatrix (San Diego, CA, USA). Genipin was obtained from Wako Chemicals (Tokyo, Japan). All other chemicals and reagents were purchased from Fisher Scientific (Waltham, MA, USA) unless stated otherwise.

### 2.2. Preparation of Electrochemical Aligned Collagen Threads

ELAC threads were prepared by following a previously published protocol with minimal modifications [6,9,12]. Briefly, monomeric acid-soluble collagen was first dialyzed by soaking 6–8 mL of solution in a dialysis tube in 2 L of ultrapure water. The water was refreshed every 2 h for the first 6 h, and the collagen was collected after 24 h of total dialysis time and stored at 2–8 °C for at least 24 h until further use. Dialysis of acid-solubilized collagen is critical for the removal of ions and allows for the formation of the pH gradient between the electrodes that drives the electrochemical alignment process via isoelectric focusing to yield ELAC threads [6]. Briefly, to fabricate the ELAC threads, dialyzed collagen was manually extruded using a syringe and a 16-gauge needle between two stainless steel electrodes of an electrochemical cell setup, and a 20 V DC voltage was applied for 60 s [6]. At the end of the process, ELAC threads were gently recovered from the electrochemical cell and incubated in 10 mL of 1× PBS for 4 h to induce gelation. The threads were then crosslinked in 10 mL of genipin solution (*w*/*v*) prepared in 90% ethanol. The experimental groups tested for ELAC included (1) uncrosslinked, (2) 0.1% genipin crosslinked for 1 h, (3) 0.1% genipin crosslinked for 4 h, (4) 2% genipin crosslinked for 4 h, and (5) 2% genipin crosslinked for 24 h, where 0.1% and 2% are genipin concentration. After crosslinking with genipin at 37 °C, the threads were washed in ultrapure water and used for further characterization.

### 2.3. Preparation of Random Collagen Threads

Dialyzed collagen and 10× PBS were combined in a 9:1 ratio, and the solution was neutralized to a pH of 7.4 using 0.1 N NaOH. The solution was thoroughly mixed, extruded into thin molds (40 mm × 1 mm × 1 mm), and incubated at 37 °C for 1 h to induce gel formation. Following gelation, random collagen was crosslinked using 10 mL of 0.1% genipin solution for 4 h at 37 °C. Uncrosslinked random collagen threads were mechanically weak for physical manipulation and, hence, were not included in this study.

### 2.4. Quantification of Crosslinking Degree of Random and Electrochemical Aligned Collagen Threads

The effect of collagen alignment and genipin crosslinking conditions on crosslinking degree was assessed by employing a Trinitrobenzene Sulfonic Acid (TNB) assay (*n* = 8/group). TNB is a spectrophotometric assay that quantifies the primary amino groups and can be used to measure the crosslinking degree by taking the ratio of the free amine groups remaining after genipin crosslinking to that present in uncrosslinked samples. Briefly, the threads were air-dried in a chemical fume hood for 12 h and the initial dry mass of the threads was recorded. The threads were then placed in a 15 mL tube to which 1 mL of 4% (*w*/*v*) sodium bicarbonate and 1 mL of 0.05% (*v*/*v*) TNBs solution were added, and the tubes were incubated at 40 °C for 2 h with a constant stirring rate of 75 rpm. Next, 3 mL of 6 M HCl was added to the tubes to prevent the TNBs from reacting further and to dissolve the collagen threads. This solution was incubated for an additional 2 h at 60 °C, and then 100 µL solution from each tube was pipetted in triplicates into a clear bottom 96-well plate. The absorbance was measured at 345 nm (Spectramax M2e, Molecular Devices, San Jose, CA, USA), and the triplicates were averaged and normalized to the initial weight of the threads to calculate the crosslinking degree. The crosslinking degree was reported as a percent of consumed primary amines, which was determined using the equation below:

(1) $$Crosslinking\;Degree=100\times \frac{1-\frac{A_{x}}{W_{x}}}{\frac{A_{N\_avg}}{W_{N\_avg}}}$$

where $A_{x}$ represents the absorbance of an averaged triplicate, $W_{x}$ represents the corresponding weight of the thread, $A_{N\_avg}$ represents the average absorbance of the uncrosslinked threads, and $W_{N\_avg}$ represents the average weight of the uncrosslinked threads.

### 2.5. Assessment of Surface Microstructure of Random and ELAC Threads

The effect of collagen alignment and crosslinking conditions on the surface microstructure of random and ELAC threads was assessed using SEM (*n* = 4/group). Post-fabrication, threads were subjected to serial dehydration using a graded series of ethanol concentrations: 20%, 50%, 75%, and 90% for 15 min each and then at 100% ethanol for one hour. Next, the threads were dried using a critical point dryer (CPD300, LEICA Microsystems, Deerfield, IL, USA), sputter coated with gold for 60 s (Denton Vacuum, LLC, Moorestown, NJ, USA), and imaged under an SEM (JEOL JSM-6380LV, Peabody, MA, USA) at 3000× magnification.

### 2.6. Mechanical Assessment of Random and Electrochemical Aligned Collagen Threads using Uniaxial Tensile Testing

Uniaxial tensile test experiments were performed to evaluate the effect of collagen alignment and genipin crosslinking conditions on the mechanical properties of collagen threads (*n* = 5–7/group). Tensile testing was performed using an HR 30 Rheometer calibrated in DMA mode (TA Instruments, New Castle, DE, USA). Briefly, collagen threads were first hydrated in ultrapure water for up to 30 min and then the threads were mounted onto the tensile grips of the rheometer with a 10 mm collagen thread gauge length. The extension rate of 5 mm/min was applied on the threads until failure. Stress was calculated by normalizing the force measurements with the cross-sectional area (calculated from the diameter of the thread and assuming a circular cross-section). Stress vs strain curves were produced, the ultimate tensile stress and ultimate tensile strain were determined, and tangential (secant) modulus [41,42] was measured by taking the slope of 3–15% strain range of the stress–strain curves to better compare the elasticity of different threads in the same strain range.

### 2.7. Statistical Analysis

Results are expressed as mean ± standard deviation. Statistical analysis was performed using one-way ANOVA with Tukey post hoc for pairwise comparisons (Prism, GraphPad, Inc., Boston, MA, USA). Statistical significance was set at *p* < 0.05.

### 2.8. Computational Modeling

**Figure 1:**
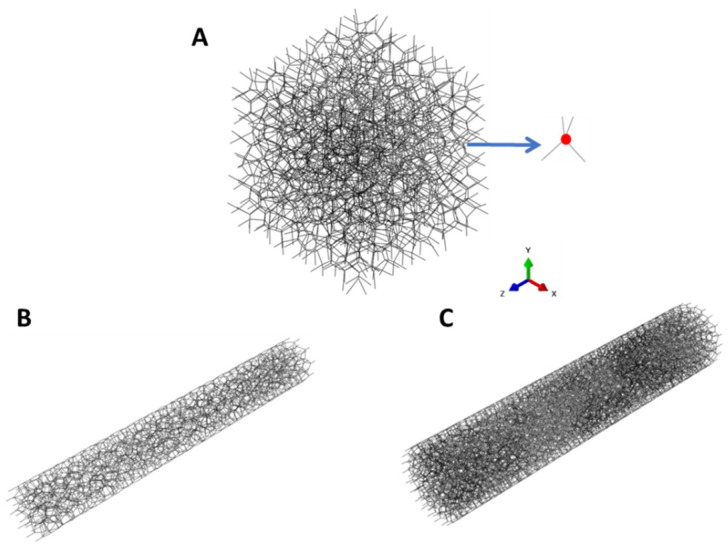
(**A**) 0.1% (4 h) random, (**B**) 0.1% (4 h) ELAC, (**C**) 2% (4 h) ELAC model.

Finite element analysis was conducted to further understand the effect of collagen fiber alignment and crosslinking degree. Three different crosslinking conditions, including 0.1% (4 h) random, 0.1% (4 h) ELAC, and 2% (4 h) ELAC threads, were chosen for finite element analysis. Three different RVEs were constructed using Voronoi network elements to reflect the microstructure and crosslinking difference in these three collagen threads (Figure 1). The Voronoi network has been validated in predicting the mechanical performance of fiber networks in our previous work [43]. In the Voronoi network, all the fibers are connected and the crosslinked parts (red dot shown in Figure 1A) are identified as the connecting points, which are connected with four individual fibers. The uncrosslinked fibers were not considered in the models since they had negligible effect on the mechanical performance of collagen threads [44]. The crosslink density is calculated as the number of crosslinked parts divided by the volume of RVE. To reflect the double crosslinking degree measured from the experiments, the crosslink density is 0.35/µm^3^ in 2% (4 h) ELAC, while it is 0.175/µm^3^ in both 0.1% (4 h) random and 0.1% (4 h) ELAC. As a result, the volume fraction of connecting fibers in random and 0.1% ELAC model is 4.3% and 8.6% for 2% ELAC model. All the individual fibers are simulated as beam elements with 0.3 µm diameter in the cross-section area. Young’s modulus is adopted as 300 MPa [45], and Poisson’s ratio is 0.3 [46]. Young’s modulus of individual fibers was kept constant for all models, as the monomeric collagen was compacted into fibrils in a short time (reduced fibril formation rate (*T*_1/2_ > 60 s)) at the beginning of the crosslinking process [47]. The crosslinking agent would not affect the fibrils’ interior structures and mechanical properties but would lead to interfibrillar crosslinking. All the RVEs were fixed at one end and stretched to 15% strain on the other end in the longitudinal direction. All the REVs were solved using the commercial software Abaqus/CAE 2019 The stress was calculated by the reaction force divided by the cross-section area of RVEs. Young’s modulus was calculated by the linear fit of the 3–15% strain range in stress–strain relationship.

**Figure 2:**
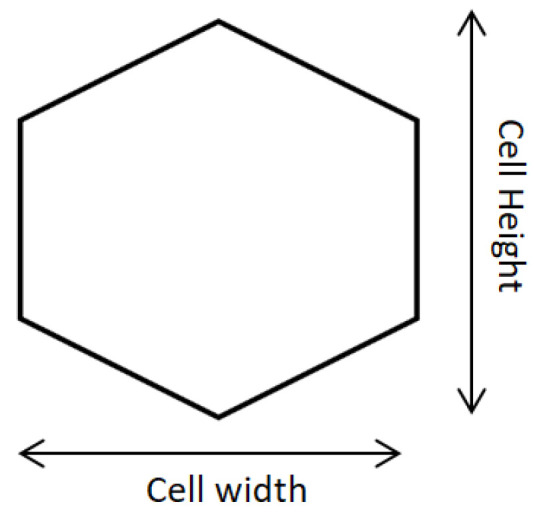
Schematic diagram of Voronoi cell illustrating cell height and width.

The electrochemically induced fiber orientation was mimicked by the aspect ratio of the Voronoi cell, i.e., the cell height divided by the cell width (Figure 2). Fiber would be more oriented to the height direction if the aspect ratio is increased. For the random crosslinking, the aspect ratio is adopted as 1 since the fibers have no preferred directions. For the 0.1% (4 h) ELAC and 2% (4 h) ELAC models, a different aspect ratio, 1.25, is adopted. The aspect ratios were set to simulate the microstructure of the composite [48].

## 3. Experimental Results

### 3.1. Quantification of Crosslinking Degree of Random and Electrochemical Aligned Collagen Threads

**Figure 3:**
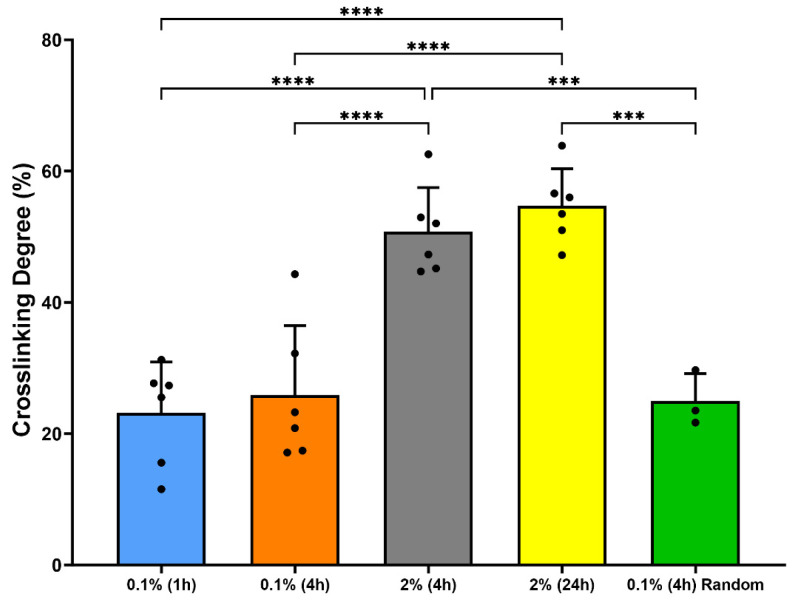
Crosslinking degree of random and ELAC threads using TNBs assay (*** indicates *p* < 0.001, and **** indicates *p* < 0.0001).

TNBs assay was used to determine the impact of genipin concentration and crosslinking duration on the degree of crosslinking of threads (Figure 3). Results showed that random and ELAC threads crosslinked with 0.1% genipin concentration exhibit a crosslinking degree of around 25%, irrespective of the duration of crosslinking. The degree of crosslinking for threads crosslinked using 2% genipin was 50–60% and comparable for both crosslinking durations of 4 and 24 h. Additionally, the crosslinking degree for threads crosslinked using 2% genipin was significantly higher (*p* < 0.001) compared to the random and ELAC threads crosslinked using 0.1% genipin for all crosslinking durations. Overall, these results indicate that the duration of crosslinking has a minimal impact on the crosslinking degree, while higher genipin concentration could significantly enhance the degree of crosslinking of ELAC threads.

### 3.2. Effect of Crosslinking Conditions on Surface Microstructure of Electrochemically Aligned Collagen Threads

**Figure 4:**
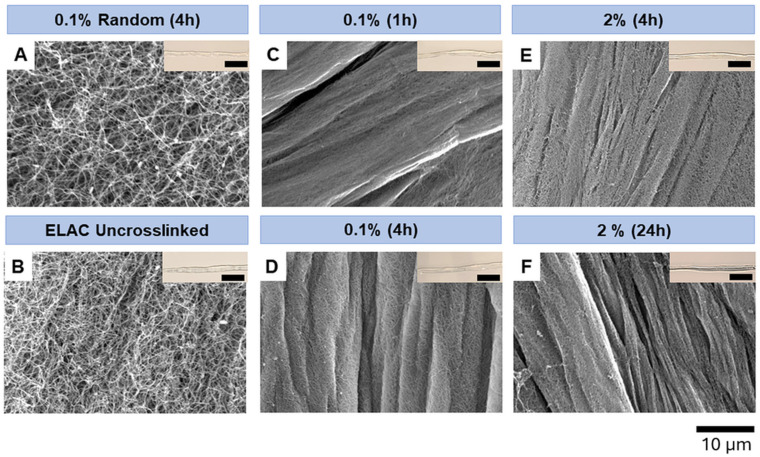
SEM images taken at a magnification of 3000× show collagen fiber alignment and packing density in (**A**) 0.1% *w*/*v* genipin crosslinked random, (**B**) Uncrosslinked ELAC, and (**C**–**F**) Crosslinked linked ELAC threads using different genipin concentrations (*w*/*v*) and crosslinking duration. Scale bar: 10 μm. Insets show an image of the actual thread (Scale bar: 5 mm).

SEM imaging was performed to determine the impact of crosslinking concentration and duration on the surface microstructure of threads. Results showed that random threads crosslinked using 0.1% genipin for 4 h displayed a classic collagen microstructure with loosely packed fibers in random orientation (Figure 4A). Uncrosslinked ELAC threads showed an aligned microstructure and resulted in the packed bundles compared to the random threads (Figure 4B). The packing of the bundles in uncrosslinked ELAC threads is looser than the crosslinked ELAC threads. All crosslinked ELAC threads showed smoother and clearer boundaries of the packed bundles (Figure 4C–F) compared to the uncrosslinked ELAC thread (Figure 4B). ELAC threads crosslinked using 0.1% and 2% genipin showed packed fiber bundles (Figure 4C–F) with greater fiber packing density in the 2% genipin crosslinked ELAC. In addition, when comparing the different crosslinking durations for 0.1% genipin, an increase in incubation time resulted in higher packing of collagen fibers.

### 3.3. Effect of Crosslinking Conditions on Tensile Properties of Random and Electrochemical Aligned Collagen Threads

**Figure 5:**
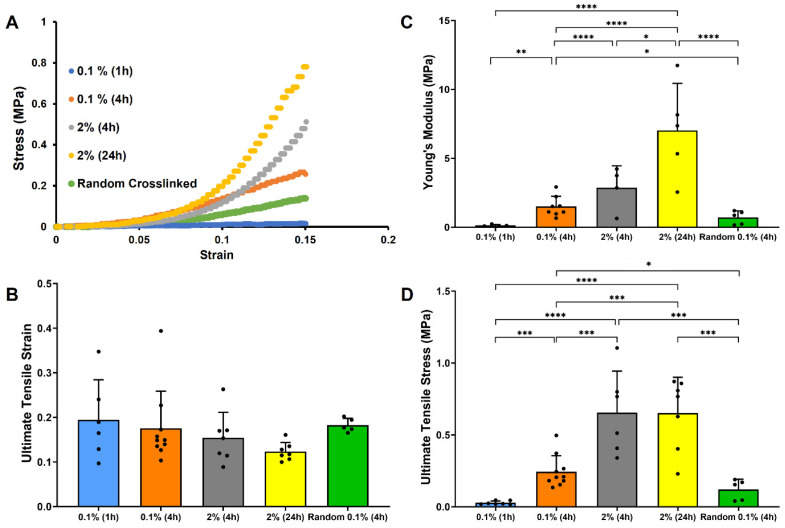
Assessment of mechanical properties of random and ELAC threads fabricated using different crosslinking parameters. (**A**) Representative stress vs. strain curves, (**B**) Ultimate tensile strain, (**C**) Young’s modulus, and (**D**) Ultimate tensile stress (* indicates *p* < 0.05, ** indicates *p* < 0.01, *** indicates *p* < 0.001, and **** indicates *p* < 0.0001).

Mechanical properties of random and ELAC threads were assessed using uniaxial tensile testing. The stress vs. strain plot showed a typically J-shaped curve with a similar initial toe region followed by a linear pattern with different slopes (Figure 5A). The slope decreased along with the decrease in crosslinking duration and genipin concentration. The Young’s modulus of ELAC threads showed a significant increase (*p* < 0.001) with an increase in crosslinking duration for both 0.1% and 2% genipin concentrations (Figure 5C). When genipin concentration increased from 0.1% to 2% for 4 hours of crosslinking, the modulus significantly increased by about 2-fold (*p* < 0.0001) because the increment of genipin concentration increases crosslinking degree (Figure 5). The modulus of ELAC and random threads crosslinked using 0.1% for 4 h was comparable, showing that the aligned fiber structure withstands more stress than the random fibers under the same strain value. Figure 5D showed that increasing the crosslinking duration from 1 to 4 h using 0.1% genipin led to an increase in the ultimate tensile stress. When comparing threads crosslinked for 4 h, increasing genipin concentration significantly increased (*p* < 0.001) the ultimate tensile stress of ELAC threads. The ultimate tensile strain shows no statistically significant difference for different genipin concentrations. Overall, with longer crosslinking duration and higher genipin concentration, ELAC threads’ Young’s modulus and ultimate tensile stress are higher.

### 3.4. Simulation Results

The Young’s modulus of ELAC threads with different aspect ratios are listed in Table 1. Random and ELAC models’ Young’s modulus increased from 0.78 MPa and 1.36 MPa to 1.53 MPa and 2.68 MPa for 0.1% (4 h) and 2% (4 h), respectively. Noteworthy, a 2% (4 h) random thread was not prepared for the experiment but was predicted in the simulation. This result indicates that the electrochemically induced fiber alignment had a great influence on the mechanical properties of collagen threads. Additionally, the crosslinking degree is another important factor affecting the mechanical properties of collagen threads. By doubling the crosslink density from 0.175/µm^3^ to 0.35/µm^3^ to represent the double crosslinking degree, Young’s modulus increased from 0.78 MPa to 1.36 MPa for random models and from 1.53 MPa to 2.68 MPa for ELAC models.

To validate the simulation results, Young’s moduli of three models were compared with the corresponding experimental measurements (Table 1). For 0.1% (4 h) ELAC and 2% (4 h) ELAC models, Young’s modulus of ELAC models matches well with the experimental measurements, indicating the fiber alignment is identical in these two collagen threads. For random crosslinked thread, the difference between experimental and simulated Young’s modulus is 9.9%. For the 0.1% (4 h) ELAC sample, the difference is 1.5%. For the 2% (4 h) ELAC sample, the difference is 6.6%.

**Figure 6:**
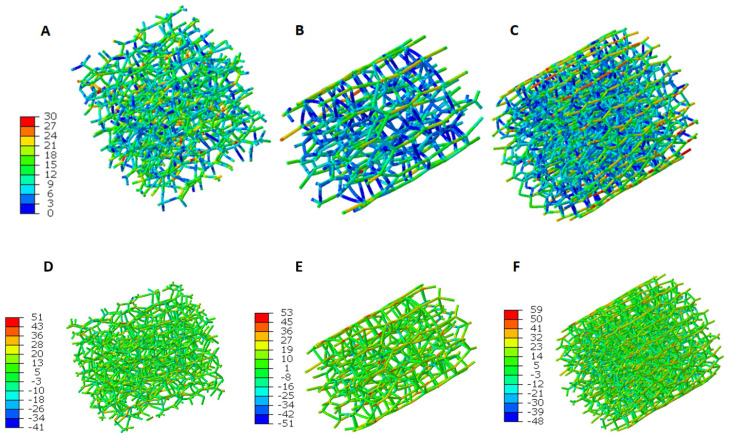
von-Mises stress (MPa) distribution of (**A**) 0.1% (4 h) random, (**B**) 0.1% (4 h) ELAC, and (**C**) 2% (4 h) ELAC models at 15% strain. Normal stress (MPa) distribution of (**D**) 0.1% (4 h) random, (**E**) 0.1%, (4 h) ELAC, and (**F**) 2% (4 h) ELAC models at 15% strain.

The contours of von-Mises stress and normal stress distribution of the central region in three RVEs are shown in Figure 6. In a random crosslinked model, the stress is widely distributed in the fiber network owing to the randomly distributed fiber structure (Figure 6A,D) because structural deformation happens in the random model, which is considered as the stretching of Voronoi cells. Compared with the random crosslinked model, the stress distribution in the other two ELAC crosslinked models shows higher values along the direction of fiber orientation (Figure 6B,C,E,F). Because structural deformation rarely happens, and the fibers in the same orientation with the load withstand more stress. Also, shear stress occurs with relatively low values for the fibers showing different orientations with the load direction, which prevents the sliding between the fibers (Figure 6B,C). Compared with the 0.1% (4 h) ELAC model (Figure 6B,E), a higher crosslinking degree contributes to a higher stress value in the 2% (4 h) ELAC model along the direction of fiber alignment (Figure 6C,F), when the overall stretch strains are the same.

## 4. Discussion

In this work, the influence of fiber alignment and crosslinking parameters on the mechanical properties of random and ELAC threads was inspected with experiments. The mechanism of the stiffness enhancement was further investigated with FEM. The crosslinking degree mainly relied on the genipin concentration, not the crosslinking duration. The crosslinking procedure leads to the formation of packed fiber bundles. Young’s modulus of the ELAC thread is related to both crosslinking degree and duration. The computational simulation illustrated that the fibers orienting in the loading direction resulted in a higher Young’s modulus under stretching. Also, the increased crosslinking degree could enhance the stiffness by increasing the shear resistance between aligned fibers during loading.

The experimental groups include the following sets: 1% (1 h) ELAC, 1% (4 h) ELAC, 1% (4 h) Random, 2% (4 h) ELAC, and 2% (24 h) ELAC. 1% (1 h) ELAC and 1% (4 h) ELAC, and 2% (4 h) ELAC and 2% (24 h) ELAC groups were set to determine the effect of crosslinking duration. 1% (4 h) ELAC and 2% (4 h) ELAC were set to determine the effect of genipin concentration. 1% (4 h) ELAC and 1% (4 h) random were set to determine the effect of collagen fiber alignment. The mechanical properties of random and ELAC threads are affected by collagen fiber alignment [23,31]. When comparing the experimental results for random threads and ELAC threads, collagen fiber alignment is the key factor affecting collagen threads’ mechanical properties. 0.1% (4 h) ELAC thread shows similar crosslinking degrees with 0.1% (4 h) random thread, but Young’s modulus for 0.1% (4 h) ELAC thread is 112.7% higher than 0.1% (4 h) random sample, and the ultimate tensile stress for 0.1% (4 h) ELAC thread is 104.2% higher than 0.1% (4 h) random sample. Previous research also demonstrated that the mechanical properties of tissue engineering scaffolds are mainly affected by the microstructures of their constitutive materials. Scaffolds composed of unidirectional constituents show higher rupture stress than multidirectional scaffolds [49].

The uniaxial tensile tests have shown that ELAC threads Young’s modulus is related to the crosslinking degree. As the crosslinking degree increased from 25% for 0.1% (4 h) ELAC to 50% for 2% (4 h) ELAC, Young’s modulus increased by 90.3%. Moreover, the crosslinking duration affected the crosslinking degree and, hence, Young’s modulus of ELAC threads. Specifically, Young’s modulus increased by 9.62 times as the crosslinking duration increased by 4 times, from 0.1% (1 h) to 0.1% (4 h) ELAC threads. The impact of crosslinking duration weakened for a higher genipin concentration. Young’s modulus for 2% (24 h) ELAC threads increased by 1.45 times compared to the 2% (4 h) ELAC threads. Modifications in the ELAC fabrication process (i.e., voltage applied, no isopropanol incubation/drying steps) employed in the current study may have yielded threads with suboptimal fiber packing density, which may explain the lower ultimate tensile stress and Young’s modulus reported in this work compared to the prior studies [23]. Another previously studied ELAC threads were crosslinked using 1-ethyl-3-(3-dimethylaminopropyl)-carbodiimide hydrochloride (EDC) and N-hydroxysuccinimide (NHS) for an overnight crosslinking treatment. The ultimate tensile stress (burst strength) was approximately 0.6 MPa for the hydrated collagen prototype [50], which is similar to the ultimate tensile stress of our 2% (4 h) ELAC and 2% (24 h) ELAC. However, we used genipin as the crosslinking agent to provide better crosslinking efficiency and cytocompatibility. Furthermore, ultimate tensile stress and Young’s modulus of patterned electrospun nanoyarn are 3.15 ± 1.56 MPa and 7.06 ± 0.95 MPa, respectively, under hydrated conditions [51]. The ultimate tensile stress is higher than our ELAC threads, but Young’s modulus is similar to the 2% (24 h) ELAC. The collagen solvent concentration was 16 *w*/*v*% for electrospun nanoyarn [51], while we used 0.31 *w*/*v*% (3.1 mg/mL) collagen solvent to manufacture ELAC threads. 2% (24 h) ELAC shows similar elasticity with electrospun nanoyarn but with a lower material consumption rate.

The computational simulation further determines the effect of crosslinking degree and fiber alignment separately at the fiber scale. With a similar crosslink degree, aligned fiber structure for 0.1% (4 h) ELAC significantly increases Young’s modulus by 96.2% compared with 0.1% (4 h) random fiber structure. As a prediction, Young’s modulus of 0.1% (4 h) ELAC and 2% (4 h) ELAC would be further increased to 1.93 MPa and 3.53 MPa with 1.4 aspect ratio, which indicates more aligned fibers showing higher Young’s modulus. The computational simulations show good results and predictions compared with the experimental data. The simulation results prove that randomly aligned fibers represent structural deformation with random stress distribution (Figure 6A). However, ELAC threads withstand more load on aligned fiber direction, with relatively lower shear stress at crosslinked parts to prevent the sliding between the fibers (Figure 6B,C). The output from the model predicts the other experiment groups well. It is not reported that we focused on genipin concentration’s influence on the crosslinking degree.

On the other hand, for the same aligned fiber structure, doubling the crosslink degree also increases the Young’s modulus. The higher crosslinking degree leads to more crosslinked parts between the fibers, which perform as stronger restrictions to withstand more shear stress [52,53]. Higher stress values can be observed near the crosslinking parts between the fibers (Figure 6C) represented by the Voronoi network.

Voronoi network acts as a close approximation to collagen gel behavior [54] with a large Poisson’s ratio (~1.5–3) [55] because of sparse distribution and low nodal degree [56]. The advantage of using the Voronoi model is represented by the unique density–moduli relationship matching with the experimental result [57]. In our study, the FEA results of the Voronoi models’ stress–strain behavior show significant agreement with the experimental Young’s modulus of the threads, which indicates the accuracy of the simulation model matching the real collagen threads based on the specific volume fraction of fiber and the Voronoi cell’s aspect ratio. With the Voronoi fiber models, the mechanical property can be predicted for random collagen thread with higher crosslinking degrees and different fiber alignments. The predicted results can be applied to customize the crosslinking parameters [58] to produce the random or ELAC threads with estimated mechanical properties.

It should be noted that the fiber network models in this study do not completely reflect all the attributes of the wide variety of fiber microstructures. The material properties of the collagen fibers were simplified as elastic materials. Hyperelasticity and viscoelasticity [59] could be considered for future studies. The Voronoi network provides a homogeneous distribution of fibers, although the distribution of collagen fibers in ELAC and random threads could be inhomogeneous [37], and the vacancies between fibers are hard to control [60]. Despite these simplifications, the feasibility of the computational method was validated by experimental testing. The current work demonstrated the impact of crosslinking degree and fiber alignment on the design and optimization of collagen scaffolds. This work can be used to provide a fundamental understanding of the intricate interplay between crosslinking parameters and mechanical performance in ELAC scaffolds. And the ELAC threads scaffold generated by optimized parameters could be further applied for tendon treatment and osteogenesis [61].

## 5. Conclusions

In conclusion, the experimental results suggest that the crosslinking degree is mainly related to the genipin concentration, not the duration of crosslinking. Crosslinking of aligned collagen fibers leads to the formation of densely packed fiber bundles. Young’s modulus of the ELAC thread is influenced by both crosslinking degree and fiber alignment. The computational simulation illustrated that fiber aligning in the loading direction resulted in a higher Young’s modulus under tensile load. And a higher crosslinking degree led to a larger stiffness due to increased shear resistance between aligned fibers. These findings could provide insights into the controlled fabrication of collagen threads. In the future, we shall design the thread with optimal Young’s modulus, uniaxial tensile stress, and uniaxial tensile strain. These ELAC threads could be prospectively used for tendon healing and replacements.

## Figures and Tables

**Table 1:** Comparison of Young’s modulus between experimental and simulation results.

	Experimental Result	Simulation Result
0.1% (4 h) ELAC	1.51 ± 0.74 MPa	1.53 MPa
2% (4 h) ELAC	2.87 ± 1.59 MPa	2.68 MPa
0.1% (4 h) random	0.71 ± 0.48 MPa	0.78 MPa
2% (4 h) random	-	1.36 MPa (prediction)

## Data Availability

The data that support the findings of this study are available from the corresponding author upon reasonable request.
